# Endoscopic removal of an impacted common bile duct stone and subsequent procedures using two devices passed simultaneously through a device delivery system

**DOI:** 10.1055/a-2915-9555

**Published:** 2026-07-29

**Authors:** Yuichi Hirata, Kazuhiro Iida, Shunsuke Nakamura, Ryosuke Mizukami, Shinsuke Akiyama, Yoshihide Ueda, Yoshihiro Okabe

**Affiliations:** 1 Gastroenterology 469536 Kakogawa Central City HospitalKakogawaHyogo PrefectureJapan

A tapered device delivery system (EndoSheather; Piolax Medical Device, Kanagawa, Japan) has been reported to facilitate the delivery of devices such as biopsy forceps and stents during biliary procedures. 1​ ​2​ Previously, we reported the removal of intrahepatic bile duct (IHBD) stones in a patient with surgically altered anatomy using the EndoSheather and a Memory Eight wire basket. 3​ However, this method had the drawback of making it difficult to leave guidewires in the IHBD, which complicated subsequent procedures. Herein, we report a case of the endoscopic removal of an impacted common bile duct (CBD) stone and subsequent procedures performed using two devices passed simultaneously through the EndoSheather.

**Fig. 1:**
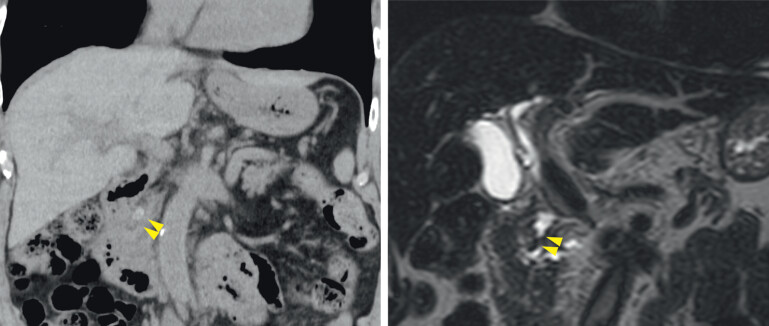
Computed tomographic and magnetic resonance cholangiopancreatographic images. CT and MRCP revealed CBD stones.

A 75-year-old man was admitted with abdominal pain and liver dysfunction. Computed tomography and magnetic resonance cholangiopancreatography revealed CBD stones ( Fig. 1 ). Endoscopic retrograde cholangiopancreatography revealed an impacted CBD stone. A conventional stone retrieval basket (FlowerBasket V; Olympus Medical Systems, Tokyo, Japan) could not be advanced sufficiently upstream of the impacted stone and instead pushed the stone proximally. Therefore, the EndoSheather was inserted deeply upstream of the stone, and only the inner cannula was removed, leaving a 0.025-inch guidewire (VisiGlide2; Olympus Medical Systems, Tokyo, Japan) in the IHBD. When only the basket wire of a lithotripsy basket (XEMEX lithotripsy basket catheter; KANEKA Medix, Osaka, Japan) was inserted alongside the guidewire within the EndoSheather, it could be delivered to the IHBD. The impacted stone was successfully removed endoscopically, and subsequent residual stone removal using a balloon catheter was performed smoothly ( Fig. 2 Video 1 ).

**Fig. 2:**
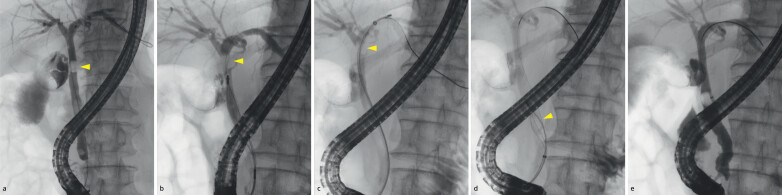
Fluoroscopic images. ( **a** ) ERCP revealed an impacted CBD stone (yellow arrowhead). ( **b** ) A conventional stone retrieval basket (FlowerBasket V) could not be advanced sufficiently upstream of the impacted stone and instead pushed the stone proximally. ( **c** ) The EndoSheather was inserted deeply upstream of the stone, and only the inner cannula was removed, leaving a 0.025-inch guidewire (VisiGlide2) in the IHBD. When only the basket wire of a lithotripsy basket (XEMEX lithotripsy basket catheter) was inserted alongside the guidewire within the EndoSheather, it could be delivered to the IHBD. ( **d** ) The impacted stone was successfully removed endoscopically. ( **e** ) Subsequent residual stone removal using a balloon catheter was performed smoothly.

**Video 1** Endoscopic removal of an impacted common bile duct stone and subsequent procedures using two devices passed simultaneously through the EndoSheather.


**Fig. 3:**
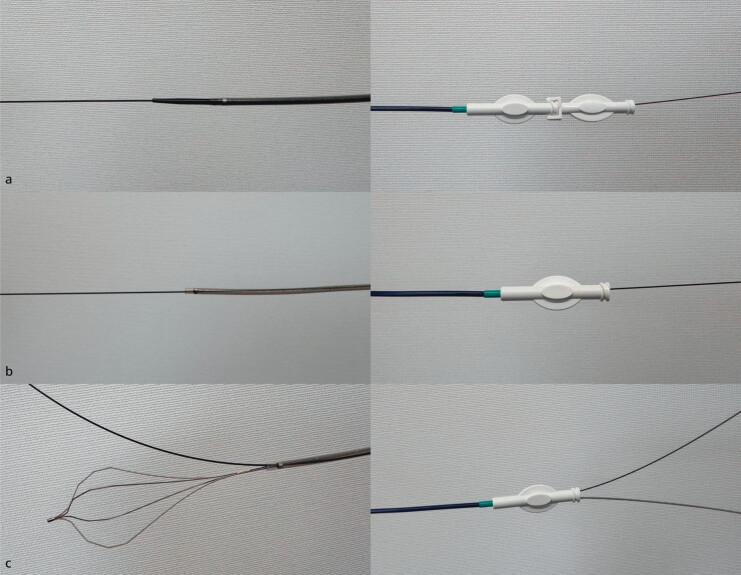
Photographs of this procedure. ( **a** ) The EndoSheather with a 0.025-inch guidewire. ( **b** ) Only the inner cannula of the EndoSheather was removed, leaving a 0.025-inch guidewire. ( **c** ) The combination of the basket wire of the XEMEX lithotripsy basket catheter and the 0.025-inch guidewire passed simultaneously through the EndoSheather.

Although many procedures using a single device via the EndoSheather have been reported, to our knowledge, this is the first report of the simultaneous use of a guidewire and a basket wire through the EndoSheather ( Fig. 3 ). The combination of the basket wire of the XEMEX lithotripsy basket catheter and the 0.025-inch guidewire passed through the EndoSheather was useful for the endoscopic removal of an impacted CBD stone and subsequent procedures.

Endoscopy_UCTN_Code_TTT_1AR_2AH
